# Staff perceptions of the management of mental health presentations to the emergency department of a rural Australian hospital: qualitative study

**DOI:** 10.1186/s12913-022-07476-7

**Published:** 2022-01-18

**Authors:** Rishaan Pawaskar, Neha Mahajan, Eklavya Wangoo, Wafa Khan, Jannine Bailey, Robyn Vines

**Affiliations:** Bathurst Rural Clinical School, School of Medicine, Western Sydney University, PO Box 9008, Bathurst, NSW 2795 Australia

**Keywords:** Emergency Department, Treatment, Mental health, Rural, Clinician Confidence

## Abstract

**Introduction:**

Current research demonstrates higher prevalence of mental health related emergency department (ED) presentations in rural areas, despite similar overall prevalence of these conditions in rural and metropolitan contexts. This stems from shortages in availability of specialised mental health professionals, greater stigma against mental illness, greater socioeconomic disadvantages, and access to means of self-harm in rural regions. Little is known, however, about the specific characteristics of mental health presentations to rural emergency departments (EDs) in Australia. Additionally, studies have shown that ED staff feel uncomfortable managing mental health presentations to ED due to factors such as lack of confidence and stigma against mental illnesses.

**Aim:**

This qualitative study sought to examine ED staff perceptions regarding the management of mental health presentations in a rural Australian ED.

**Methods:**

A qualitative study design was used, incorporating semi-structured interviews of current ED staff. Ten interviews were conducted in person or over the phone by two researchers and thematically analysed to draw out key themes from the data.

**Results:**

Staff perceived deficiencies in availability of mental health expertise, de-escalation, and referral pathways as major barriers to effective patient management. These factors contributed to increased retention of mental health patients in ED due to uncertainties regarding their definitive care. Despite acknowledging the value of practical experience with mental health presentations as the best way of increasing clinician confidence, staff expressed a desire for more face-to-face training to better equip them to respond to mental health presentations.

**Conclusion:**

A combination of departmental and hospital-wide issues in conjunction with individual staff attitudes regarding mental health conditions contributes to issues in mental health patient care in this ED. In particular, limited training in mental health and resources available to ED staff affects confidence in managing mental health presentations and contributes to prolonged time to definitive treatment.

## Introduction

Approximately 23% of individuals living in the state of New South Wales (NSW), Australia have an underlying mental health condition [1], with 17.7% of adults aged 16 and over experiencing high or very high levels of psychological distress in 2019 [2]. The prevalence of mental health conditions is similar between metropolitan and rural areas [3], however, higher rates of self-harm and suicide are found in rural areas [4], with a significant proportion of completed suicides involving gun use [5].

Hospital emergency departments (EDs) can often be the first point of health service contact for individuals with mental health conditions [6]. In 2015-16, approximately 3.7% of all Australian public hospital ED presentations were due to a primary mental health condition [7]. Statistics show higher rates of ED presentation per 10,000 population in rural and remote areas [4]. Proposed reasons for this include: a shortage of specialised mental health professionals in rural areas [4]; differing help-seeking behaviours in rural areas particularly due to high levels of stigma in smaller communities [4, 5]; greater socioeconomic disadvantage, access to means of self-harm and greater social isolation in rural and remote locations [8]; and the impact of environmental factors such as prolonged droughts which contribute to mental illness and higher self-harm rates [4].

Given such factors promoting the higher prevalence of mental health presentations to rural EDs, it is important to evaluate the expertise and resources within EDs to accommodate this demand. Regarding management in ED, generally fast-paced ED environments may adversely impact on patients with mental health presentations. An Australian report indicated that mental health presentations to EDs often involved longer waiting times compared to other presentations and they were more likely to leave the ED against medical advice [9]. Potential reasons include the complexity of mental health presentations and the perception of these requiring less urgent interventions, thereby delaying treatment provision [10]. A study on the attitudes of ED staff towards mental health presentations found that some considered ED inappropriate for the treatment of mental health patients, due to noise levels and relative lack of privacy [10]. Personal prejudices regarding mental health conditions also potentially impact care provided [10]. One qualitative study found greater staff negativity towards these presentations, less inclination to provide help, and perception of mental health presentations as “ingenuine” [ 10, 11]. Comparing rural to urban EDs, an Australia-wide study of ED clinicians found views of poorer mental health patient care and prejudicial attitudes in rural areas [12]. This was attributed to lower education and training opportunities and discrepancies between available staff and workload within ED [12].

Over the preceding decade, rural NSW has experienced a prolonged drought, proceeded by a bushfire crisis, and more recently, the Covid-19 pandemic. The compounding impacts of these events on the mental health and wellbeing of regional communities is acknowledged [13]. It is therefore timely to examine the preparedness of rural EDs to respond to mental health presentations. This study sought to examine the perceptions and attitudes of ED staff regarding care of mental health presentations at a rural hospital in NSW, Australia.

## Methods

### Study design & ethics

A qualitative study design was employed. Ethics approval was obtained from the Greater Western Human Research Ethics Committee (HREC; 2019/ETH116131) and the Western Sydney University HREC (H133831).

### Recruitment & data collection

Eligible participants were those clinicians employed within a rural hospital ED during the study period (June 2019 to March 2020). Participants were recruited by email invitation and by study promotion during ED team meetings. Interviewees participated in semi-structured, face-to-face or telephone interviews conducted by Authors 1-4. The interviewers may have been known to the interviewees as they were 5^th^ year medical students undertaking their rotations at this hospital at the time of data collection. The interviewers were trained in qualitative data collection procedures by investigators 5 and 6, to ensure consistency in data collection.

Interviews were audio-recorded and transcribed verbatim. Interviews last for 15 to 30 minutes and consisted of four open ended questions and related probes to elicit further details (Table 1). The interview schedule was developed by the researchers based on a previous survey of Australian ED clinicians [14], and adapted to the study setting.

**Table 1:** Interview schedule for clinicians regarding management of mental health presentations to a rural emergency department

Questions	Question probes as needed
1. How do you feel about managing mental health related presentations to the emergency department?	a. *What is your level of confidence and what factors affect this?* b. *What could improve your confidence?* c. *Do you have any concerns regarding the management of mental health patients, particularly in a rural setting?*
2. How would you describe the adequacy of the resources and referral pathways available in emergency department for mental health patients?	d. *What resources would you like to see increased or made available?* e. *Are there any issues with use of existing resources e.g. safe rooms*
3. How would you describe the training you have received so far in managing mental health presentations to emergency department?	f. *What specific training have you received and has it been adequate?* g. *Is further training easily accessible to you? What barriers might you encounter in accessing further training?*
4. What is your opinion on the management and transfer of scheduled patients to [nearby centre], particularly given the 2015 changes requiring ambulance escort rather than police transfer? How has the conversion of this hospital’s emergency department to a gazetted facility affected patient management?	h. *What are the benefits of this system?* i. *Are there any issues associated with the system?* j. *Has this affected the care of non-mental health patients in the ED?*

### Data analysis

De-identified interview transcripts were analysed by Authors 1, 2 and 4 under the guidance of Authors 5 and 6. Close reading of the transcripts prior to commencing analysis ensured familiarisation with the data. The first cycle of coding was used to generate a preliminary codebook which was discussed and reviewed. The codebook was refined through subsequent cycles of coding until a consensus was reached regarding the emergent themes [15].

### Study context

The rural hospital ED which was the site of this study typically has about 1200 mental health presentations per year in an average 20,000 total presentations. The majority of mental health presentations are voluntary and the most common presentation is suicidal ideation/self-harm. The majority of all presentations are managed and discharged within ED without admission. The ED has three mental health clinical liaison nurses who are rostered on from Monday to Friday from 8:00am to 9:00pm, and on weekends from 8:30am to 4:30pm. After hours mental health support is available through the Mental Health Care Emergency Care Rural Access Program which is accessed by telehealth linkup to a nearby facility.

## Findings

### Clinician perspectives on mental health presentations to ED

Ten participants were interviewed; eight nurses and two doctors. The nurses had worked in the ED for three or more years while the doctors had worked in the ED for at least eleven weeks each year, as per their hospital terms. Five main themes and several subthemes were identified as summarised in Table 2. Exemplar quotes for each theme are provided in Tables 3, 4, 5 and 6 and are labelled according to participant type (nurse or doctor) and number.

**Table 2:** Summary of themes and subthemes identified from the interviews with clinicians regarding management of mental health presentations in the emergency department

Theme	Subtheme
Clinician perceptions of mental health patients in ED	*○ Mental health patients in ED as disruptive and a cause of stress/concern* *○ Impact of stigma on mental health patient care*
Challenges in current management practices in this ED	*○ Mental health patient management is a challenge in terms of balancing efficiency, security, patient care* *○ Defensive practice of medicine* *○ Inappropriateness of ED in mental health patient management* *○ Views of safe room in ED* *○ Breadth of mental health and the issues it creates with triage of mental health patients* *○ Limitations of current referral pathways and limited after hours support* *○ Long wait times for definitive management for mental health patients in ED*
Importance of mental health expertise availability in this ED	*○ Lack of mental health expertise in ED affects system efficiency* *○ Critical need for mental health liaison nurse in the ED* *○ Afterhours patient management* *○ Need for on-call psychiatrist*
ED staff experience of training in mental health	*○ Practical experience with mental health patients improves confidence* *○ Desire for more in-hospital training at this facility* *○ Lack of mental health training at this facility* *○ Barriers to further training in mental health*

**Table 3:** Subthemes identified for the main theme – clinician perceptions of mental health patients in ED

**A. Mental Health patients in ED as disruptive and a cause of stress/concern**	
*I have concerns about mental health patients who are involuntary, who are say booked to go to [acute facility], who spend a lot of time in ED. I find them –The ones who are in the normal beds, there’s cords, there’s all sorts of stuff around, and that makes me a bit nervous. [Nurse 1]* *There have been patients where they have been like psychotic and running around naked and that can be pretty disruptive. Because of that it’s a lot more stressful for the nurses. [Doctor 1]* *It’s just moving them through the ED into like a better – for the particularly aggressive and psychotic patients- into like a safer environment for everyone, because they are really disruptive. [Doctor 2]* *Is benefit outweighing the risk.... If you put an acute mental health patient next to a two-year-old toddler and the mental health patient’s swearing, and cussing, and carrying one, it’s less than ideal. [Nurse 4]*	
**B. Impact of stigma on mental health patient care**	
*There are certainly people who have a bad attitude towards all mental health, but I think when it comes down to it, I think in this department mental health patients get treated with the compassion and respect they deserve. The attitude is there, but I don’t think the attitude necessarily negatively affects treatment of patients. So, I don’t know if I’d say it’s a problem that needs addressing [Nurse 1]* *I do definitely think that like – and I’m a victim to it too, but just the stigma around mental health. It does affect you and then it affects the care for the patient. [Doctor 1]* *[With] drug and alcohol issues, and socioeconomic issues, or Indigenous issues, race and things like that... they [other staff] might judge people for what they do instead of being helpful.[Nurse 6]*

**Table 4:** Subthemes identified for the main theme – Challenges in Current Management Practices in this Rural ED

**A. Mental health patient management is a challenge in terms of balancing efficiency, security, patient care**	
*There’s a different approach you have to take and especially with mental health, definitely a type of emotional attention… A lot of times just being in the ED it’s because you’re balancing some of the other patients, it’s hard to give that emotional attention. I find it difficult the line between having to clear the patient medically and then also being helpful and therapeutic for the actual mental health problem difficult. [Doctor 1]* *It’s not necessarily difficult to deal with, but it’s very, very time consuming. If you’re doing a ten hour night shift and you’ve got four patients and one of them is a mental health patient who is scheduled and can’t leave, and you get busy with your three medical type patients – normally we’ll have security there, but even so you don’t really know how much of an eye security is keeping on them- and you never know what they might get up when you’re not there watching them. [Nurse 1]* *I think one minute they say we shouldn’t tolerate any aggression then on the other side, we’ve got to accept and try and tolerate these sorts of people with aggression because it’s a patient…And the amount of security is that – some of the guys are useful, but some of them are not really used to doing any physical restraining and that’s really hard. [Nurse 8]*	
**B. Defensive practice of medicine**	
*Whereas if you have one of our doctors who sees a patient and – a lot of the time a lot of it can be kind of arse covering…If they have a suspicion that this person might go off and do something that they may be culpable in the long run they won’t hesitate to say ‘Uphold the section. Refer them on to MHEC [mental health emergency care]’. And, then this person who might just be having some sort of situational crisis and said a few things that they didn’t mean…they end up spending eight hours in ED waiting for a plan. [Nurse 1]* *Not many doctors are willing to do them [form 1] because once they revoke them, they then have to deal with if that person goes out and does something to themselves or others. So, not many clinicians will actually do the form 1 or are willing to do it in our department. [Nurse 8]* *The big problem is afterhours...in the middle of the night are you going to let someone go home that’s just come in and said I don’t feel safe to be at home? No, you’re not. It doesn’t matter whether you’re a mental health trained person or not, you’ve got to think about patient safety at the end of the day. We’ve got a duty of care, regardless of whether you’re mental health or not. [Nurse 5]*	
**C. Inappropriateness of ED in mental health patient management**	
*They [patients] feel that, just being in the ED and with the ED it makes them worse. It’s a lot of waiting for things. It’s hard to avoid. [Doctor 1]* *And it makes it hard for the rest of the department because there’s kids in there, there’s elderly, they have to hear all this yelling and the abuse, you know the screaming and also if the patient is scared, they’ve got to listen to all that and I don’t think it’s healthy for them too. [Nurse 8]* *19-year-old girl who breaks up with her boyfriend and then locks herself in the bathroom and threatens to kill herself- when you have a full department and no beds and nowhere to put anyone, and you’ve been flogged all night, and then that person comes in with police screaming their head off and carrying on, that makes me kind of roll my eyes a little bit and just go ‘Do I really have to?’ [Nurse 1]*	
**D. Views of safe room in ED**	
*If I had my way, I would always keep – anyone who you’re even a little bit suspicious about kind of suicidal/homicidal, anything at all - I would always keep them in the mental health room, because there’s nothing in there that you can fiddle with, you know. [Nurse 1]* *I think more isolation rooms would probably be quite useful and just that sort of privacy…I feel like they still like a bit of confidentiality. [Doctor 2]* *It is a suitable room to have a private conversation with someone. I think that all the mental health patients should be going in there…But when you’re on a bed you’ve got no privacy. [Nurse 3]* *It still is effectively two gym mats that you’re sleeping on in a safe assessment room. So to have patients unnecessarily in there overnight…because of staffing issues …I think that’s pretty poor that we don’t have something better. [Nurse 2]* *A lot of the time after hours I just park them in there, [thinking] I’m too busy…Because you’re in there and you’re behind, you’re not in the visual field, you do sometimes get forgotten. [Nurse 5]* *I’ve had a lot of patients; they feel like they’re in prison in that room. [Doctor 1]*	
**E. Breadth of mental health and the issues it creates with triage of mental health patients**	
*Generally, a lot of things are triaged now as mental health... I had a patient yesterday which had an acute behavioural disturbance, but it was in the context of intellectual disabilities and it really wasn’t a mental health presentation at all, but that’s what they triaged as and it still warrants that assessment. [Nurse 2]* *So if you’ve got someone who’s 70 years old, has never had a psychiatric history, no recent trauma, and they’re presenting in a confused, perplexed, acute sort of phase, most people go, “Oh, it’s metal health because they’re seeing things, they’re hearing things”, but nine times out of ten it’s a delirium. [Nurse 2]* *The umbrella of mental health is big. The umbrella of drug and alcohol is big…you’ve just got a bit of anxiety, but in the big scheme of things you fall under the same umbrella and have to be treated exactly the same, where you need a lot more resources than what you do. [Nurse 5]*	
**F. Limitations of current referral pathways and limited after hours support**	
*Because they’re all private… we can direct admit people from ED to there if we have an accepting doctor, and then our ward doctor, the ward doctor from this hospital, will go and review patients in that unit. [Nurse 1]* *Because Community [mental health] only work 9:00 to 5:00 type hours. It’s all afterhours stuff that it gets a bit tricky. [Nurse 1]* *…there’s a period of time when there’s no cover and then the evening person comes on. [Doctor 1]* *No support afterhours. That’s a massive limitation where after hours you’re relying on that 1300 number to give you advice on how to best manage this patient. Where if it was a cardiac patient you ring up the cathlab in [town], but mental health, you just can’t ring a psychologist up…You need a specialist to guide you, that’s where that all breaks down. [Nurse 5]* *The biggest thing that we have trouble doing is from here, getting them to [acute facility] if they need that. They can wait here for days and that’s not good for them because they’re usually in a little confined area and hear the ‘beep, beep, beep’ of the alarms. It’s not the right place for them to be. They’ve got to start here of course but it’s just the timeframe to get them up there which can stop them. It could be transporters, it can be bedlock. Bedlock’s a huge problem and there’s more people with mental health issues now. [Nurse 7]*	
**G. Long wait times for definitive management of mental health patients in ED**	
*Sometimes our mental health patients can sit there for three-four hours, depending on how busy MHEC is. There are some nights where you have four or five people who self-present as voluntary people seeking help or whatever, and you have to sit them in the waiting room and say ‘It may be a couple of hours until someone in [MHEC] is ready to talk to you’. It’s not ideal. [Nurse 1]* *There are no beds available anywhere else and no one’s willing to accept them and move them on from the ED, and then that does create an issue of like inadequate resourcing because you don’t have enough rooms for people, but that’s just more, I guess a general issue. [Nurse 5]* *They wait no longer than any other person to see a general specialist… If you walked around the unit today the average stay overnight, we’re at 16 and 17 hours for patients overnight within the ED to actually get a bed, get transferred, have that review by the specialist service. [Nurse 5]* *For a definitive answer, probably yes, maybe. Or for a definitive transfer time they definitely are [waiting longer]. [Nurse 3]* *We quite often will get them reviewed quite quickly. It’s the waiting for that secondary review and them saying let them sleep overnight and get them followed up by the [mental health clinical liaison nurse], that’s an ongoing concern that they’re pushing back to the resources that are here. [Nurse 4]*	

*MHEC* Mental Health Emergency Care (An after-hours telephone/videoconference service)

**Table 5:** Subthemes identified for the main theme – availability of Mental Health expertise in the ED

**A. Lack of Mental Health expertise in ED affects system efficiency**	
*I think it reassures the patient as well, being seen by someone who is a mental health worker…They can give them a bit of an idea about what’s going on, whereas for the most part, all we tell them is ‘We’ll wait for the interview at [nearby regional centre]’. [Nurse 1]* *There’s no psych registrar, no psychiatrist, no-one on the ground that can assist... so when I have to come back and make the calls, having someone that can sit with mental health consumers, be with them and talk to them and calm them down, that would be helpful … If there was a mental health social worker that we have access to that we could sort of - weekends and after hours, we have a lot of presentations for D&A come in. It sort of lands back on mental health…and patients have to wait. [Nurse 2]* *Designated mental health workers in EDs are an absolute essential because they understand the schedules, the legal requirements. They are constantly educating us on how we’re supposed to go about a job and guiding and directing us as to where we’re going, and the junior doctors as well. [Nurse 4]*	
**B. Critical Need for Mental Health liaison nurse in the ED**	
*I think there’s a big gap when there’s no liaison nurse, no mental health liaison nurse. When they’re not here, there’s a massive gap in the mental health services that we provide. In essence, we provide none. [With the MHCL] the big difference if that you have a plan much much earlier. [Nurse 1]* *I think because they’re so qualified in mental health and de-escalation. They set a tone and they give such guidance with their level of experience and exposure, understanding their patient history. [Nurse 4]* *We’ve actually got three of them [MHCLs] that are brilliant and they’re very quick at picking up things … I really appreciate their input and they’re very experienced. [Nurse 7]* *Because they’re [MHCL] a specialist in that field. They’ve got the resources. They’ve got the contacts. It’s like me talking to ICU, or me talking to admin. I know exactly who I’ve got to talk to, what I’ve got to do, what I’ve got to do to get that resource. Same with the mental health side of things. [Nurse 5]*	
**C. Afterhours patient management**	
*It’d be nicer to have them [MHCL] a bit longer rather than have to get onto the video link-up because when you get onto the video link-up, you have to wait. You have to get in a queue because they [MHEC-RAP] do a third of the state. [Nurse 7]* *They’re [MHEC-RAP] not always available immediately to do assessments, patients have to wait, patients get annoyed with waiting. [Nurse 2]* *Sometimes our mental health patients can sit there for three/four hours, depending on how busy MHEC is. And, you can imagine having an agitated, maybe paranoid– trying to reassure someone like that for four hours…it’s very, very difficult … they’re all waiting for the same camera in the same room with the same person based out of [nearby regional centre] [Nurse 1]* *It’s harder to get them assessed afterhours, especially on evening or night shift when there’s no cover, or weekends. [Nurse 6]* *There are people who work in the Department who are really great in assessing patients but afterhours it’s not so great in terms of staff and referral especially when you’re facing them [mental health patients] more then. [Doctor 2]*	
**D. Need for on-call psychiatrist**	
*The buck stops here kind of thing … with no psychiatrist seeing them. I talk to them on the phone and they’re going purely off what I’ve seen, so it’s not like they’re seeing the patient… so that to me is concerning. [Nurse 2]* *Because they could see the patients and assess them here and then they wouldn’t need to be transferred anywhere, they could deal with them here and go home or something. [Nurse 3]* *No because the psychiatrist themselves is never going to come and see the patient. You still need your liaison nurse to physically see the patient. [Nurse 1]* *We sort of do with the telephone. Would I love it if we had one here? Absolutely. Would it be an appropriate use of resources? No. When you consider the presentations of MH compared to medical, we couldn’t justify having a psychiatrist on site here, not just for the ED, possibly in the community section. [Nurse 4]*	

*MHEC* Mental Health Emergency Care (An after-hours telephone/videoconference service), *MHEC-RAP* Mental Health Emergency Care Rural Access Program

**Table 6:** Subthemes identified for the main theme – ED Staff Experience of Training in Mental Health

**A. Practical experience with mental health patients improves confidence**	
*Experience I think (improves confidence in managing mental health patients), it’s taken a long while umm, years. [Nurse 8]* *While I think that I have had a lot of teaching … there’s nothing like actually going in there and having the experience to deal with it. [Doctor 1]*	
**B. Desire for more in-hospital training at this Facility**	
*There is online stuff available…But I think more in-services for nurses particularly from mental health nurses who actually know...more stuff around the legal side of it and the different kinds of involuntary patients and maybe some stuff around doing brief mini MSEs [Mental State Examinations]. [Nurse 1]* *It would be good to have just more tutorials on managing them acutely in the ED. It’s a bit different to seeing them in the acute mental health like ward when you’re doing this. [Doctor 2]* *When you do it online it’s not the same as if you have someone face-to-face, give you a bit more skills and case studies and things like that that they’ve had experience with, things that we could do better. [Nurse 6]* *I’ve had a lot of ED experience, I’ve been here for more than 12 years, but I still don’t feel 100% confident. I could definitely do with more training and face-to-face, online, more in-services and things like that, more up to date information. [Nurse 6]* *I think face-to-face education is an enormous challenge. I always believe that face-to- face education is so much more valuable than online. [Nurse 4]* *We deal with frontline, but we don’t deal with communicating with them and how to do a proper assessment, and aggression minimisation or any other techniques, calming techniques and things like that would be handy. [Nurse 6]* *They’ve been trying to run violence prevention and management training for some time. The big challenge that we have with that is that quite often the dates will be released well after the time the roster’s done, so it makes it very difficult for management. [Nurse 4]*	
**C. Lack of mental health training at this Facility**	
*I’ve received no mental health training whatsoever from this hospital at all. [Nurse 1]* *You learn it at uni, but in terms of on the job kind of refreshers and things like that, you don’t do any of it here. [Nurse 1]* *We get our tutorial sessions and that’s the extent of it. [Doctor 2]*	
**D. Barriers to further training in Mental Health**	
*It always comes down to money and can we relieve you, can we get someone to backfill your position while you do that, that is a huge factor…if it’s not directly related to your area or your specialty, it might not be approved. [Nurse 2]* *(There is) not much training in emergency mental health, so locally, obviously, and you sort of look at your circumstances outside of work and find that balance of how much time you spend away training as well. I’m not in a position to sort of go off to [city] for a week and do a course. [Nurse 2]* *Online learning is a necessary evil... A couple of staff that have really struggled on farms and things that don’t have good internet, I’ve given them study leave days, allocated them an office and tried to get their study leave done online. [Nurse 4]*	

#### Clinician perceptions of mental health patients in ED

Participants described varying perceptions of mental health patients in the ED. For some, they described mental health patients as disruptive and a cause of stress because of the unpredictable, and potentially unsafe, behaviours they were concerned would occur with mental health patients. For others, this was seen as par for the course in ED and that the behaviours of mental health patients were not dissimilar to those of a confused patient or a patient with dementia (Table 3a).

The impact of staff perceptions on mental health patient care was also reported variably. Whilst some participants reported that negative perceptions (should they exist) do not impact patient care, others thought that such negative perceptions do negatively impact patient care (Table 3b).

#### Challenges in current treatment practices in this rural ED

The current elements of mental health patient care at this ED were discussed by participants, including both broader departmental level and individual staff-patient level issues. Mental health patient care was described as a tug-of-war between efficiency and comprehensive patient care, and at the same time, between duty of care and defensive medical practice. The type of mental health presentation negatively affected many staff experiences due to the need for increased monitoring and resource allocation for risky or disruptive patients. This was particularly challenging when there were already high patient loads in ED and this challenge is exacerbated due to the inconsistency in the quality of security services available in ED to assist clinicians (Table 4a).

The particular challenges associated with mental health related presentations were reported by participants to lead to the adoption of a defensive clinical practice by many clinicians in the ED. Due to the perceived potential risk that mental health patients may represent to themselves and the community if discharged, participants reported a perception that clinicians prolonged the ED stay to prevent themselves being culpable for a mental health patient’s behaviour (Table 4b).

The ED was considered by participants as an inappropriate setting overall for mental health patient care for the reasons outlined above. The impact on mental health patients themselves of being in the ED environment was considered to be negative and conversely the impact of mental health patients on other patients in the ED was also viewed as being negative (Table 4c).

The safe room in this ED was regarded as an essential component in caring for mental health patients, particularly those with potential for self-harm. It was considered a means of improving mental health patient privacy within the busy ED environment. The safe room however was also used for detaining mental health patients whilst awaiting transfer to a nearby facility, and thus carried the risk of deprioritising mental health patients and contributing to stigmatisation by virtue of separation (Table 4d).

Participants described the triage of patients with situational issues, drug and alcohol or acute behavioural disturbance under mental health as inappropriate, and a contributing factor to both overutilization of resources and staff frustration. This was partly attributed to the broad spectrum of mental health conditions and the difficulty in differentiating psychiatric disorders from acute medical problems such as intellectual disability and delirium, which can mimic depression and psychotic states (Table 4e).

The difficulty in accessing referral pathways was also highlighted, especially afterhours due to the lack of availability of MHCLs (Table 4f). This contrasted with medical patients in ED, for whom clearly defined, and easily accessible specialist medical guidance was consistently available. Additionally, local mental health facilities were not accessible to ED staff for referral of mental health patients requiring further evaluation, as these were privately run (Table 4f).

Long waiting times for definitive management is a common issue raised (Table 4g). Several participants reported wait times in excess of 3-4 hours for a video link after hours when a MHCL was unavailable for face-to-face consultation within the ED. Patients often waited in the ED safe room overnight for review the next morning, which was difficult logistically and distressing for these patients. Some participants however attributed long wait times to a hospital-wide systemic issue, indicating them to be no different to long wait times experienced by medical patients (Table 4g).

#### Availability of mental health expertise in the ED

Availability of staff with adequate mental health experience and expertise was judged an important factor impacting on patient care in this ED. A lack of staff skilled in the treatment of MH patients was reported by participants, which in turn prolonged waiting times for definitive care and contributed to greater staff workloads (Table 5a). Additionally, staff with expertise in mental health case management were reported to be useful for other ED staff in improving understanding and application of the legalities around mental health patients in ED, whilst also being able to enhance the experience for patients themselves (Table 5a). Regarding available staff, participants highlighted the importance of the ED’s MHCL in improving mental health patient care and resource management in the ED as they assisted with earlier provision of management plans (Table 5b). Afterhours patient management, when availability of MHCLs or other similar staff is limited, was considered a major problem by participants, compounding patient waiting times, ED patient load and overall inefficiency of the system (Table 5c). Some participants discussed the need for an on-call psychiatrist, favouring this for improved patient care (Table 5d). In contrast, however, other participants found minimal additional value in the presence of an on-site psychiatrist as their role carried the potential for resource inefficiencies, being essentially fulfilled by the MHCL.

#### ED staff experience of training in mental health

Practical experience with mental health presentations was reported as a significant contributor to the level of confidence amongst ED staff. In particular, staff reported that regardless of their background education, it was exposure to mental health patients in hospital that improved their confidence with these cases (Table 6a).

Despite acknowledging practical experience as the best method of increasing confidence, participants also expressed a desire for more in-hospital training particularly face-to-face (Table 6b). Suggestions included training about legal issues, acute management of mental health presentations, and de-escalation techniques for agitated patients.

The majority of participants agreed that opportunities for mental health training were limited at this facility (Table 6c). Where training has been provided onsite, challenges with late scheduling of training dates and conflicts with ED rosters were noted (Table 6b-c). Key barriers to out-of-hospital training were the cost for the facility, both in terms of the actual training cost and the cost of providing backfill to someone’s role, and balancing work-life commitments (Table 6d). One participant noted that even the completion of mandatory online modules had been difficult for staff members with poor internet access. Overall, most participants expressed a desire to seek further education and training in mental health if resources and support were more easily accessible.

## Discussion

This study aimed to identify clinician perceptions of mental health patient care in a rural ED. The core findings of this study were that perceived deficiencies in staff expertise, de-escalation techniques and referral pathways contributed to increased mental health patient retention in ED, thereby increasing staff workloads.

Staff at this ED felt under-trained in responding to mental health presentations, and deficient in their understanding of mental health legislation, de-escalation techniques for agitated patients, and confidence in caring for acute presentations. They encounter several barriers to accessing adequate mental health training outside of hospital to enable them to upskill, including costs, lack of backfill for their role, work-commitments, poor internet access and rural location. This is consistent with the findings of two Australian studies regarding clinicians’ confidence in responding to mental health presentations to rural EDs [16, 17]. To mitigate these factors, participants in our study requested more traditional ‘face-to-face’ teaching in-hospital as this would overcome the need to travel to access training and thereby reduce the amount of time they need away from the ED to upskill. A 2020 Australian qualitative study found that a single four-hour simulation-based educational workshop run by, and for, ED staff members improved understanding of mental health legislation and patient treatment [18], thereby providing a potential strategy to improve staff confidence.

In this ED, clinicians deferred to their MHCLs for responding to mental health presentations, due to their greater training and experience in assessment of and provision of treatment plans and pathways. However, the MHCL was not available after hours in this ED. An extensive body of research attests to the valuable role a MHCL can play in an ED setting providing timely, specialist mental health support for patients [19–23]. The importance of consistent staffing availability for such as role has also been noted in metropolitan research [24]. Participants in this study reported similar benefits of their MHCLs. A key barrier for providing effective mental health care in this ED was cited as the lack of availability of their MHCLs after hours to facilitate local referrals and quick assessment and care of mental health presentations. However, it is still vital for all ED staff to be equipped with the skills to respond to mental health related presentations. Poorer ED staff confidence in mental health conditions can potentially lead to delayed definitive management and contribute to overall greater patient and resource loading within the ED [25].

Increased retention of mental health patients is further compounded by systemic barriers within ED, such as inaccurate triage, overcrowding and an unsuitable physical environment. These barriers contribute to inefficient resource allocation, defensive clinical practice and create a potentially distressing patient experience, leading some participants to question the appropriateness of ED for mental health care. Difficulty in triaging mental health patients via the Australasian Triage Scale was raised by participants, and has similarly been reported in surveys of ED clinicians [26, 27]. This is partially attributable to insufficient training and difficulty in applying standard medical triage protocols to mental illness [26]. Uncertainty regarding triage severity may lead to underestimation of triage categories, increased workload and inefficient resource allocation [26], with subsequent reliance on available specialist mental health expertise or a defensive approach to provision of care until such expertise is available.

Many participants reported employing defensive practices in mental health patient care, such as unnecessary hospital admission or refusal to discharge patients, as a response to inadequate skills, insufficient psychiatric expertise and concerns regarding clinician safety and liability. Defensive medicine is a common practice amongst ED clinicians, supported by a study of psychiatric nurses and psychiatrists in New Zealand [28], which found that concerns regarding patient risk to self and others led to delayed discharge and hospital admissions. A study of ED nurses in a Western Australian hospital, further suggests that defensive attitudes may escalate potentially violent patients [17]. Participants highlighted that the noisy environment of ED and lack of privacy may be potentially distressing to mental health patients, and added to difficulties in patient assessment. This must be considered in this ED where seventy-three percent of presentations in 2018 were treated and discharged within ED without admission. Studies have suggested that such busy ED settings and associated sensory overstimulation may limit the appropriateness of ED in mental health patient management [29]. This raises the question of appropriate safe room use for patient assessment, a topic on which our participants were divided. Some felt that safe rooms exacerbated the issue of deprioritisation, while others felt a safe room was essential for patient safety and privacy. Overall, systemic barriers and delays in management of mental health patients are co-existing issues that affect the efficiency of patient management in the ED.

The combination of limited resources, inadequate referral pathways and inefficient patient transport culminates in delays in patient transfer and definitive management. Our study identified a significant shortage of specialist psychiatric services and guidance for ED clinicians, particularly overnight/after hours. Despite the implications of inaccessible specialist psychiatric expertise, participants were divided on the need for an on-site psychiatrist, as the theoretical benefits did not seem to balance the costs in an already busy ED. As discussed previously, participants believed increased MHCL staff coverage after hours would improve wait times, reduce patient load, and minimise backlog for offsite mental health assessment units. One study demonstrated that ED clinicians experience greater confidence in treating mental health patients when specialist mental health staff, such as MHCL nurses, are based in the ED overnight [30]. These factors, in combination with delays in patient transport and transfer by ambulance, have contributed to the long wait times for definitive management experienced by mental health patients. Australian findings show that mental health patients consistently wait longer than patients with physical illness for assessment and treatment, and have longer durations of stay in ED, a finding also noted here. With many of the mental health presentations to this ED involving suicidal ideation or self-harm, these wait times are concerning.

We acknowledge certain limitations to our study. Our sample consisted predominantly of nurses, with only a few junior doctors participating and no senior doctors, which may limit the applicability of our findings to a general population of ED clinicians. Nonetheless, data saturation was deemed to have been achieved with this participant group. It is also noteworthy that it is typically the nurses and junior doctors on the ground who provide the hands on patient care in this ED setting. Therefore, while we were not able to capture the perspectives of some of the senior clinicians, our data provide insight into the perspectives of those staff at the coalface delivering care to mental health patients. Future research could attempt to directly link clinician perspectives with presentations they are responding to in real time such as with a clinician survey completed after each encounter. This would enable us to directly link specific characteristics of each presentation with the particular barriers encountered in this ED setting during the provision of care. At the same time, the perspectives of mental health patients could also be examined to determine their experiences of care in the ED. Our clinician participants noted the limited availability of community services for mental health care in this rural location. By engaging directly with patients, this would enable us to delve further into the specific barriers they encounter with seeking support and care in the primary healthcare setting and community setting in this rural location. As this was a case study at a single site, future research should also seek to compare with other rural locations and with urban locations to determine which issues are specific to this rural location and which are more widespread in our healthcare system.

## Conclusion

A combination of departmental and hospital-wide issues, in conjunction with individual staff attitudes regarding mental health conditions, contributes to issues including increased time to definitive treatment of mental health patients in this rural ED. Previous studies have highlighted negative staff attitudes towards mental health conditions and concerns regarding the appropriateness of ED in responding to such presentations. However, this study demonstrates specific challenges facing rural EDs including staff undertraining in mental health, inadequate MHCL coverage and inconsistencies in triage and available referral pathways. These factors lead to greater departmental and resource loading and may incite negative views of mental health presentations amongst ED clinicians due to work disruption and exposure to aggressive patients.

## Data Availability

The datasets used and/or analysed during the current study are available from the corresponding author on reasonable request.

## Abbreviations

ED: Emergency Department
HREC: Human Research Ethics Committee
MH: Mental Health
MHCL: Mental Health Clinical Liaison Nurse
NSW: New South Wales
